# Occurrence of Stigmatizing Documentation Among Hospital Medicine Encounters With Opioid-Related Diagnosis Codes: Cohort Study

**DOI:** 10.2196/53510

**Published:** 2024-10-24

**Authors:** William S Bradford, Reed W R Bratches, Hollie Porras, David R Chen, Kelly W Gagnon, Simon B Ascher

**Affiliations:** 1 Division of Infectious Diseases University of Alabama Birmingham Birmingham, AL United States; 2 School of Nursing University of Alabama Birmingham Birmingham, AL United States; 3 Department of Pharmacy University of California Davis Medical Center Sacramento, CA United States; 4 Department of Internal Medicine University of California Davis Medical Center Sacramento, CA United States; 5 School of Public Health University of Alabama Birmingham Birmingham, AL United States

**Keywords:** stigmatizing language, OUD, opioid use disorder, people with opioid use disorder (POUD), people who inject drugs (PWID), people who use drugs (PWUD), drug use

## Abstract

**Background:**

Physician use of stigmatizing language in the clinical documentation of hospitalized adults with opioid use is common. However, patient factors associated with stigmatizing language in this setting remain poorly characterized.

**Objective:**

This study aimed to determine whether specific demographic factors and clinical outcomes are associated with the presence of stigmatizing language by physicians in the clinical documentation of encounters with opioid-related *ICD-10* (*International Statistical Classification of Diseases, Tenth Revision*) codes.

**Methods:**

Hospital encounters with one or more associated opioid-related *ICD-10* admission diagnoses on the hospital medicine service during the 2020 calendar year were analyzed for the presence of stigmatizing language in history and physical and discharge summaries. Multivariable adjusted logistic regression models were used to determine associations of age, race, gender, medication for addiction treatment use, against medical advice discharge, homelessness, comorbid polysubstance use, comorbid psychiatric disorder, comorbid chronic pain, cost, and 30-day readmission with the presence of stigmatizing language.

**Results:**

A total of 221 encounters were identified, of which 64 (29%) encounters had stigmatizing language present in physician documentation. Most stigmatizing language was due to use of “substance abuse” rather than the preferred term “substance use” (63/66 instances). Polysubstance use and homelessness were independently associated with the presence of stigmatizing language (adjusted odds ratio [aOR] 7.83; 95% CI 3.42-19.24 and aOR 2.44; 95% CI 1.03-5.90) when controlling for chronic pain and other covariates.

**Conclusions:**

Among hospital medicine encounters with an opioid-related diagnosis, stigmatizing language by physicians in clinical documentation was common and independently associated with comorbid polysubstance use and homelessness.

## Introduction

The opioid epidemic is fueling a massive rise in inpatient admissions related to opioids, with a 3.2-fold increase in these hospitalizations between 1998-2000 and 2015-2016 [1] and an accompanying approximate 4.6-fold increase in opioid-related deaths over the same time period [2]. Patients who misuse opioids experience substantial stigma in the health care setting [3-5], which is associated with numerous deleterious effects including increased rates of patient-directed or against medical advice (AMA) inpatient discharge, delays in health care, and undertreatment of pain and withdrawal symptoms [6]. The University of New South Wales Stigma Indicator Tracker noted 1-year rates of witnessed externalized stigma among health care workers toward people who inject drugs to be 70%, which is higher than stigma based on sexual orientation, sex work, or HIV status [7].

Growing recognition that stigma is highly prevalent has led to calls for increased research activity to understand the drivers of stigma [8] as well as larger-scale health policy reform [9]. The “Words Matter” guidance document from the National Institute on Drug Abuse (NIDA) recommends avoiding the use of certain phrases viewed as stigmatizing in favor of person-centered language in the health care setting [10]. However, diagnostic documentation practices lag, with current English *ICD-10* (*International Statistical Classification of Diseases, Tenth Revision*) codes still reflecting stigmatizing language, such as “drug abuser” and “opioid abuse” [11]. The potential impacts of existing stigmatizing language in diagnostic documentation are compounded by the 21st Century Cures Act that requires patients to have access to their health record. Facilitated by the increase in electronic health records with patient portals, it is necessary to identify groups with a higher likelihood of stigmatizing language in clinical documentation [12].

In this study, we sought to identify associations between stigmatizing language in physician documentation and key sociodemographic factors and substance use–related outcomes characteristics. We hypothesized that the presence of stigmatizing documentation would be associated with decreased odds of addiction-related care delivery, increased odds of AMA discharge, and increased odds of 30-day readmission.

## Methods

### Data Source

The University of California Davis Medical Center is a 646-bed tertiary care center serving northern California and western Nevada. It has an active addiction medicine consult service as well as an opioid stewardship pharmacy service. During the study period, internal medicine physicians staffed the hospital medicine service with no resident learners, scribes, or other intermediaries regularly involved in documentation production. The hospital medicine group was medium in size (staff size 47-54), exclusively allopathic internal medicine trained, and mostly recently graduated from residency (42%-55% of the group was <5 years out of residency over the course of the year). There were no specific treatment protocols in place pertaining to treatment of opioid use disorder, although the general expectation was that the addiction medicine consult service would be involved. We included all patients admitted to the hospital medicine service and hospitalized for more than 24 hours from January 2020 to December 2020 with an *ICD-10* code consistent with opioid use (refer to Multimedia Appendix 1 for a list of all *ICD-10* codes used).

### Chart Review

A nonblinded attending physician reviewer [WB] and nonblinded clinical pharmacist reviewer [HP] manually audited patient charts. Sociodemographic information was obtained by electronic health record auto-populated data (Table 1). Physician-completed history and physical and discharge summary documentation was manually screened by one of the authors [WB] for evidence of stigmatizing language as determined by the NIDA “Words Matter” educational material [10]. When present, the first encountered instance of stigmatizing language encountered was recorded as a “specific term.” If multiple instances of stigmatizing language were very close to one another in the medical chart (eg, within the same sentence), both were recorded. Data were recorded in a spreadsheet format using Microsoft Excel 365.

**Table 1:** Descriptions and data sources of variables.

Variable		Source	Description
**Sociodemographics**			
	Age	Vizient	Age at time of admission
	Race	Vizient	Race, patient-reported during registration
	Gender	Vizient	Gender, patient-reported during registration
	Homelessness	Vizient	Homelessness status, as determined during registration
**Comorbidities**			
	Psychiatric diagnosis	Manual abstraction	Presence of posttraumatic stress disorder, bipolar disorder, schizophrenia, schizoaffective disorder, major depressive disorder, or other major psychiatric disorder
	Polysubstance use	Manual abstraction	Evidence of use of other substance (eg, cocaine and methamphetamine)
	Chronic pain diagnosis	Manual abstraction	Evidence of any chronic pain-related diagnosis associated with the encounter
**MAT^a^**			
	MAT POA^b^	Manual abstraction	Evidence of prescribed buprenorphine, methadone, or naltrexone use at time of admission
	MAT initiation	Manual abstraction	Evidence of prescribed buprenorphine, methadone, or naltrexone use at time of admission
**Outcomes**			
	Cost	Vizient	Total billed cost of encounter
	AMA^c^	Vizient	Whether encounter had an against medical advice-type discharge
	30-day readmission	Vizient	Whether the patient had another inpatient encounter within 30 days or now

^a^MAT: medication for addiction treatment.

^b^POA: present on admission.

^c^AMA: against medical advice.

### Relevant Measures

Covariates (Table 1) were selected by the study team based on their clinical expertise and knowledge. Covariates included age, gender, race, 30-day readmission, total cost of hospitalization, AMA discharge, comorbid polysubstance use, comorbid major psychiatric diagnosis, comorbid chronic pain diagnosis, medication for addiction treatment (MAT) presence on admission, and MAT initiation during inpatient stay. The primary outcome was presence of stigmatizing language, which was defined as the presence of at least 1 instance of stigmatizing language as defined by the NIDA “Words Matter” document in the physician’s clinical documentation during the hospital encounter [10].

### Statistical Analysis

Univariate analysis was conducted between the outcome and the covariates using the independent samples *t* test and chi-square test as applicable. In multivariable models adjusting for age, race, and comorbid chronic pain, logistic regression was used to determine the associations of each covariate of interest with the presence of stigmatizing language in the electronic health record. Collinearity between covariates was evaluated using the variance inflation factor, and problematic collinearity was defined as a variance inflation factor of greater than 10 [13]. We did not impute or otherwise include responses with missing data. Analyses were conducted using R software (version 3.4.1, R Foundation for Statistical Computing).

### Ethical Considerations

This study was reviewed by the institutional review board of the University of California Davis and ruled to be not human participants research. Study data were securely stored on the institutional OneDrive storage platform until data collection was complete, then all patient identifiers were removed for this secondary analysis.

## Results

### Overview

A total of 221 encounters met the inclusion criteria. The mean participant age was 51, 101 of 221 (46%) were female, 113 of 221 (51%) White, 44 of 221 (35%) Black, and 145 of 221 (28%) unhoused (Table 2). Comorbid polysubstance use (92/221, 42%), chronic pain (111/221, 50%), and major psychiatric disorders (75/221, 34%) were common. Readmission within 30 days occurred following 47 of 221 (21%) of encounters, and AMA discharges occurred in 24 of 221 (11%). Stigmatizing language was present in 64 of 221 (29%) of the evaluated encounters, with 63 of the 66 observed occurrences being “abuse” in preference to “use” and a small number due to “clean” or “dirty” (2 of 66) or “Intravenous drug user” (1 of 66).

**Table 2:** Variables by stigmatizing language status with accompanying univariate and bivariate analyses.

Variables			No stigmatizing language (n=157)		Stigmatizing language (n=64)		Total (N=221)		*P* values
**Age**									.40
	Mean (SD)	51.4 (16.7)		49.4 (15.6)		50.9 (16.3)			
	Range	19.2-88.9		26.5-80.5		19.2-88.9			
**Cost**									.18
	Mean (SD)	143515 (241511.524)		100982 (100578.091		131198 (211259.313)			
	Range	6140-2605565		13090-582756		6140-2605565			
**Gender, n (%)**									.12
	Female	77 (49.0)		24 (37.5)		101 (45.7)			
	Male	80 (51.0)		40 (62.5)		120 (54.3)			
**Race, n (%)**									.10
	White	78 (49.7)		35 (54.7)		113 (51.1)			
	Black	61 (38.9)		16 (25.0)		77 (34.8)			
	Other	18 (11.5)		13 (20.3)		31 (14.0)			
**30-day readmission, n (%)**									.02
	No	117 (74.5)		57 (89.1)		174 (78.7)			
	Yes	40 (25.5)		7 (10.9)		47 (21.3)			
**AMA^a^, n (%)**									.054
	No	144 (91.7)		53 (82.8)		197 (89.1)			
	Yes	13 (8.3)		11 (17.2)		24 (10.9)			
**Homelessness, n (%)**									<.001
	No	115 (82.1)		30 (48.4)		145 (71.8)			
	Yes	25 (17.9)		32 (51.6)		57 (28.2)			
**Polysubstance use, n (%)**									<.001
	No	114 (72.6)		15 (23.4)		129 (58.4)			
	Yes	43 (27.4)		49 (76.6)		92 (41.6)			
**Psychiatric diagnosis, n (%)**									.48
	No	106 (67.5)		40 (62.5)		146 (66.1)			
	Yes	51 (32.5)		24 (37.5)		75 (33.9)			
**MAT^b^ POA^c^, n (%)**									.52
	No	110 (70.1)		42 (65.6)		152 (68.8)			
	Yes	47 (29.9)		22 (34.4)		69 (31.2)			
**MAT initiation, n (%)**									.007
	No	148 (94.3)		53 (82.8)		201 (91.0)			
	Yes	9 (5.7)		11 (17.2)		20 (9.0)			
**Chronic pain diagnosis, n (%)**									<.001
	No	57 (36.3)		53 (82.8)		110 (49.8)			
	Yes	100 (63.7)		11 (17.2)		111 (50.2)			

^a^AMA: against medical advice.

^b^MAT: medication for addiction treatment.

^c^POA: present on admission.

### Bivariate and Multivariable Analysis

In unadjusted bivariate analysis, polysubstance use (*P*<.001), MAT initiation during an encounter (*P*=.01), and homelessness (*P*<.001) were associated with presence of stigmatizing language, while chronic pain was significantly less likely to co-occur with stigmatizing language (*P*<.001). Other demographics and clinical characteristics were not associated with stigmatizing language (Table 2).

Due to missing data related to homelessness, only 202 encounters were able to be included in the multivariable model. In multivariable models, polysubstance use and homelessness were independently associated with the presence of stigmatizing language (adjusted odds ratio [aOR] 7.83; 95% CI 3.42-19.24 and aOR 2.44; 95% CI 1.03-5.90, respectively). Chronic pain was significantly associated with the absence of stigmatizing language (aOR 0.24; 95% CI 0.10-0.55). Other variables, including race and rates of MAT use, were not significantly associated with stigmatizing language (Table 3).

**Table 3:** Multivariable-adjusted associations of patient demographics and clinical characteristics with stigmatizing documentation.

Predictors		aOR (95% CI)	*P* values
Age		0.99 (0.96-1.02)	.44
**Race**			
	Black	0.78 (0.30-1.97)	.60
	Other	1.54 (0.51-4.61)	.44
Gender, male		1.72 (0.77-3.87)	.19
Homelessness		2.44 (1.03-5.90)	.04
Polysubstance use		7.83 (3.42-19.24)	<.001
Psychiatric diagnosis		0.62 (0.26-1.43)	.28
Chronic pain diagnosis		0.24 (0.10-0.55)	.001
MAT^a^ POA^b^		1.12 (0.48-2.59)	.79
MAT^a^ initiation		0.99 (0.33-2.98)	.99
AMA^c^		1.09 (0.34-3.48)	.88
Cost		1 (1.00-1.00)	.87
30-day readmission		0.77 (0.23-2.35)	.65

^a^MAT: medication for addiction treatment.

^b^POA: present on admission.

^c^AMA: against medical advice.

## Discussion

### Principal Results

In this single-center study of 221 hospital encounters with an opioid-related *ICD-10* diagnosis on the hospital medicine service, use of stigmatizing language in physician documentation was common. However, most stigmatizing language identified involved “[substance] abuse” rather than more overt forms of stigmatizing language catalogued in NIDA’s “Words Matter” educational material, such as “Intravenous drug user,” “clean” or “dirty.” We found that polysubstance use and homelessness were independently associated with the presence of stigmatizing language when controlling for chronic pain and other covariates, while other demographics and clinical characteristics had no significant associations. Significantly, rates of MAT initiation, AMA discharge, cost, and 30-day readmission did not differ significantly between the 2 groups, indicating that the use of stigmatizing language did not have a measurable effect on these major outcomes.

### Limitations

Our study had several limitations. First, our study sample was derived from a single hospital medicine service at an academic medical center with a strong addiction medicine service in a state with established access to postdischarge MAT, which may have different documentation and treatment practices than other less-resourced centers. Second, we made use of 2 reviewers who did not overlap their review of charts. While they were experienced chart reviewers (an attending physician and opioid stewardship pharmacist), multiple overlapping reviews would have been optimal. In addition, we were unable to report the number of unique patients included in the study due to the way patient health information was archived. Finally, “[substance] abuse” is a set of *ICD-10* diagnostic codes, so although use of the term is considered stigmatizing language, it is consistent with current medical coding practices and may not necessarily reflect enacted stigma in its typical usage, potentially reducing the external validity of the study.

### Comparison With Previous Work

Our research supports the idea that stigmatizing language in clinical documentation is associated with meaningful differences in patient characteristics. These differences may result in or reflect enacted stigma and thereby impact patient outcomes. Past work has indicated that stigmatizing language evokes more negative judgements among health professionals compared with the use of person-centered language [14,15]. Patients also prefer when their providers use of person-centered language [16], indicating language shift may be a low barrier opportunity to improve patient trust. A recent study among hospitalized patients with infectious complications of opioid use showed that having a documented plan for ongoing treatment and addiction-specific follow-up are associated with the presence of best practice documentation [17]. They similarly found that stigmatizing language was common and mostly involved use of “abuse” rather than the preferred term “use” or “use disorder.” Detection and real time intervention on stigmatizing language in mental health care may be aided using natural language processing (NLP) [18]. A large study using an NLP algorithm trained to detect stigmatizing language found similar specific language and participant sociodemographic associations to our study [19].

### Conclusions

Stigmatizing language in documentation of opioid-related encounters is common and independently associated with homelessness and polysubstance use but not any major substance use-sensitive outcomes like MAT use, MAT initiation, AMA discharge, or 30-day readmission. Use of stigmatizing language in clinical documentation has critical implications for people who use opioids who access their health records; to address their medical mistrust and experienced stigma, providers should strive to use best practice documentation. NLP approaches may allow for better detection of this language for research and clinical improvement purposes. The next revision of *ICD-10* should eliminate use of “[substance] abuse” in favor of current best practice nonstigmatizing documentation in accordance with “Words Matter” guidance. Future studies that prospectively assess implementation strategies to reduce use of stigmatizing language in documentation would be useful.

## Appendix

Multimedia Appendix 1 Additional methods 1.

## Abbreviations

AMA: against medical advice
aOR: adjusted odds ratio
ICD-10: International Statistical Classification of Diseases, Tenth Revision
MAT: medication for addiction treatment
NIDA: National Institute on Drug Abuse
NLP: natural language processing
