# Supplementary material for: Occurrence of Stigmatizing Documentation Among Hospital Medicine Encounters With Opioid-Related Diagnosis Codes: Cohort Study
Source: JMIR Form Res. 2024 Oct 24;8:e53510. doi: 10.2196/53510 (PMC11544335; doi:10.2196/53510)
Supplement: Multimedia Appendix 1 [file formative_v8i1e53510_app1.docx]

**Supplemental Methods: ICD-10 Codes Utilized**

- - **Poisoning by ____ (all types of opioids), initial or subsequent encounter**. T40.0X1A,T40.0X4A,T40.1X1A,T40.1X4A,T40.2X1A,T40.2X4A
  - **Opioid abuse with various remission and current use states** F11.10,F11.11,F11.120,F11.121,F11.122,F11.129,F11.14,F11.150,F11.151,F11.159,F11.181,F11.182,F11.188,F11.19
  - **Opioid dependence with various remission status and complications statuses**

F11.20,F11.220,F11.221,F11.222,F11.229,F11.23,F11.24,F11.250,F11.251,F11.259,F11.281,F11.282,F11.288,F11.29

- - **Opioid use, unspecified with various complications**

F11.90,F11.920,F11.921,F11.922,F11.929,F11.93,F11.94,F11.950,F11.951,F11.959,F11.981,F11.982,F11.988,F11.99
